# Coronary computed tomography angiography findings of anomalous left hepatic vein drainage into the coronary sinus with complex anomalies: a case report

**DOI:** 10.1093/ehjcr/ytag487

**Published:** 2026-07-18

**Authors:** Ömer Baran Yaman, Dursun Can Karakuş, Turgut Seber

**Affiliations:** Department of Radiology, Kayseri City Hospital, Şeker Mah. Muhsin Yazıcıoğlu Bulvarı No:77, Kocasinan, Kayseri 38080, Turkiye; Department of Radiology, Kayseri City Hospital, Şeker Mah. Muhsin Yazıcıoğlu Bulvarı No:77, Kocasinan, Kayseri 38080, Turkiye; Department of Radiology, Kayseri City Hospital, Şeker Mah. Muhsin Yazıcıoğlu Bulvarı No:77, Kocasinan, Kayseri 38080, Turkiye

**Keywords:** Case report, Coronary sinus, Left hepatic vein, Coronary computed tomography angiography, Partial anomalous pulmonary venous return, Persistent left superior vena cava

## Abstract

**Background:**

Drainage of the left hepatic vein into the coronary sinus is an extremely rare anomaly reported in the literature. Typically, haemodynamics remain unimpaired in the absence of concomitant anomalies, and detection is expected to be incidental.

**Case summary:**

We herein report a case of a 7-year-old male patient presenting with atypical chest pain, exertional dyspnoea, and mild congestive hepatomegaly, in whom a left hepatic vein draining into a dilated coronary sinus was detected. This anomaly was associated with a superior–inferior type sinus venosus atrial septal defect, persistent left superior vena cava, and partial anomalous pulmonary venous return. Together, these defects created a significant left-to-right shunt, explaining the patient's symptomatic right ventricular strain.

**Discussion:**

This case underscores the diagnostic necessity of computed tomography angiography in evaluating complex venous anatomy and its clinical implications. Our findings demonstrate that when echocardiography fails to fully explain right-sided volume overload and atypical symptoms, advanced cross-sectional imaging is decisive for a comprehensive anatomical diagnosis and multidisciplinary surgical planning.

Learning points

- Unexplained right-sided heart dilation on echocardiography should always prompt comprehensive cross-sectional imaging to rule out rare concomitant venous anomalies and shunts.
- Coronary computed tomography angiography is an excellent, non-invasive modality for precisely delineating complex congenital venous malformations.
- Accurate pre-operative mapping of an anomalous left hepatic vein draining into the coronary sinus is essential for planning surgical cannulation strategies and preventing intraoperative complications.

## Case summary

A 7-year-old male patient presented to the paediatric cardiology outpatient clinic with complaints of atypical chest pain, mild exertional dyspnoea, and easy fatigability. Physical examination revealed a normal physiological status without any prominent pathological murmurs; however, the liver was palpable 2 cm below the costal margin, suggesting mild congestive hepatomegaly. Laboratory findings, including complete blood count and cardiac biomarkers, were within normal limits. Initial electrocardiogram demonstrated a right axis deviation, consistent with right heart strain. Transthoracic echocardiography revealed a dilated right atrium and right ventricle with preserved left ventricular systolic function. A sinus venosus type atrial septal defect (ASD) and suspected partial anomalous pulmonary venous return (PAPVR) were also noted.

However, because standard echocardiography has limitations in visualizing posterior venous structures, it could not fully explain the severity of the right heart dilation and hepatomegaly. Therefore, a contrast-enhanced coronary computed tomography angiography (CCTA) was performed. CCTA confirmed that the right heart and pulmonary artery were markedly dilated. It definitively revealed a partial anomalous pulmonary venous return, with the right superior, middle, and inferior pulmonary veins draining into the right atrium, alongside a superior–inferior type sinus venosus ASD (*Figure 2*). Furthermore, an anomalous left hepatic vein and a persistent left superior vena cava were observed draining into a dilated coronary sinus (*Figure 1*).

**Figure 1:**
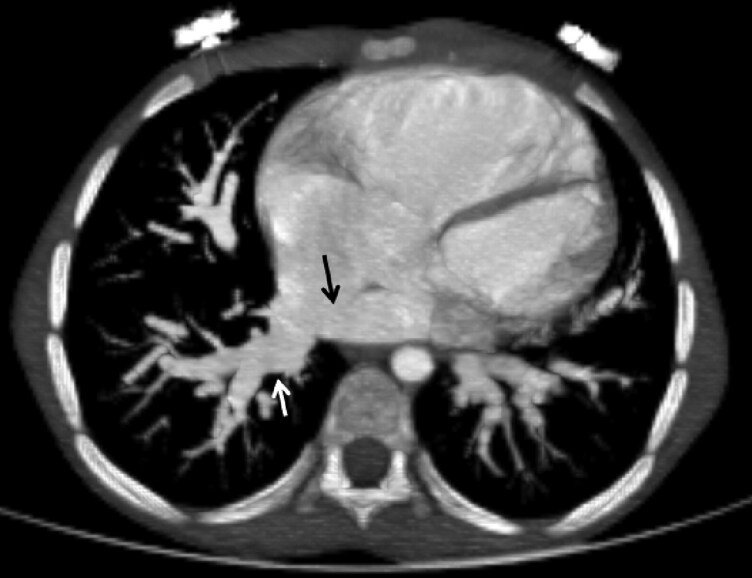
Axial coronary CT angiography (MIP) images demonstrating a sinus venosus type ASD (black arrow). The right upper and middle pulmonary veins (white arrow) merge at the ostial level, forming a common trunk that drains into the right atrium. ASD, atrial septal defect; MIP, maximum intensity projection.

**Figure 2:**
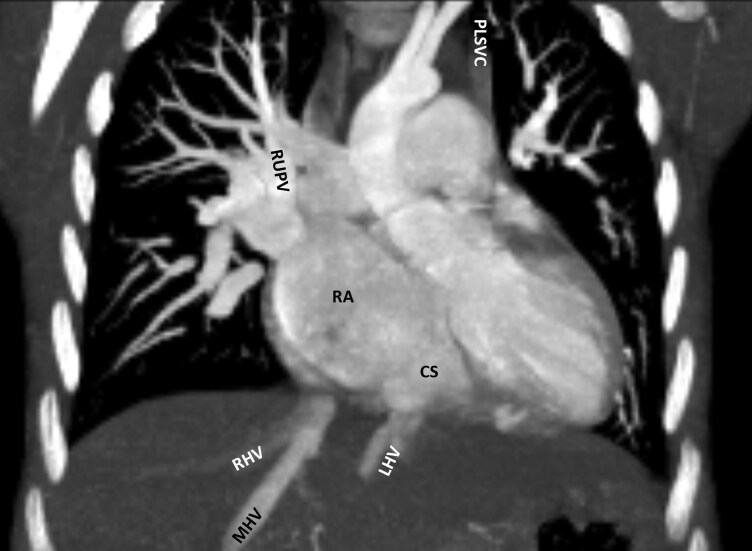
Coronal MPR MIP coronary CTA images demonstrate the left hepatic vein draining into a dilated coronary sinus. Additionally, a persistent left superior vena cava is present. The right upper and middle pulmonary veins merge proximal to the ostium, draining into the right atrium via a common trunk. RMPV, right middle pulmonary vein; RUPV, right upper pulmonary vein; PLSVC, persistent left superior vena cava; RA, right atrium; CS, coronary sinus; RHV, right hepatic vein; MHV, middle hepatic vein; LHV, left hepatic vein.

Following the comprehensive radiological evaluation, a multidisciplinary paediatric cardiology and cardiovascular surgery council was planned to discuss the optimal management strategy, including potential surgical correction of the ASD and anomalous venous connections. However, as the patient was a foreign national, he was unfortunately lost to follow-up due to geographical relocation before a definitive therapeutic intervention could be initiated.

## Discussion

Direct drainage of the left hepatic vein into the coronary sinus is an extremely rare anomaly. It arises from the persistence of the connection between the left vitelline vein and the coronary sinus during embryological life.^1–3^ Nearly all variations of the left hepatic vein are associated with the right hepatic vein, with 61% draining via a common trunk into the inferior vena cava, and 39% draining via separate trunks into the inferior vena cava.^4^ In the literature, the drainage of the left hepatic vein into the right atrium or the coronary sinus is limited to only a few reported cases. When other anomalies are present, they frequently include conditions such as ASD or PLSVC (*Table 1*). When isolated, it generally does not impair haemodynamics.^3,6^ Therefore, it is detected incidentally. As such, most reported cases are in adults or found in autopsy series.

**Table 1.** Previously reported cases of anomalous left hepatic vein drainage in the literature and associated anomalies

Study (ref)	Hepatic vein anomaly	Age/sex	Associated anomalies	Clinical presentation	Diagnostic modality
Buehler *et al*.^5^	LHV to CS	19/F	PLSVC	Dyspnoea	Not reported
Karolczak *et al*.^2^	LHV to CS	2/M	ASD, APVR	Not reported	Not reported
Rajesh *et al*.^6^	LHV to CS	28/F	ASD (Ostium secundum), PLSVC	Not reported	MRA
Lee *et al*.^3^	LHV to CS	61/F	None	Congestive heart failure	Cardiac CT
Morshuis *et al*.^7^	LHV to CS	76/F	None	Incidental	Intraoperative
Milisavljevic *et al*.^8^	LHV to RA	21/M	PLSVC	Not reported	Dissected specimen (Autopsy)
Suzuki *et al*.^9^	LHV to CS	72/F	None	Not reported	Intraoperative
Vuran *et al*.^1^	LHV to CS	6/M	ASD (Perimembranous)	Headache	Doppler US

LHV, left hepatic vein; CS, coronary sinus; PLSVC, persistent left superior vena cava; ASD, atrial septal defect; APVR, anomalous pulmonary venous return; RA, right atrium; MRA, magnetic resonance angiography; CT, computed tomography; US, ultrasound.

Among adults, ASD has an incidence of 0.13% in the USA. Four types of ASD exist, with the most common being the ostium secundum defect. The rarest is the sinus venosus ASD. The sinus venosus type is subdivided into superior and inferior types. Sinus venosus ASD accounts for 5–6% of all defects.^10^ The vast majority of sinus venosus ASDs are accompanied by anomalous pulmonary venous return.^11^ In our case as well, the partial anomalous pulmonary venous return, in which the right superior, middle, and inferior pulmonary veins drained into the right atrium (*Figures 2 and 3*), was accompanied by a large superior-inferior type sinus venosus ASD.

**Figure 3:**
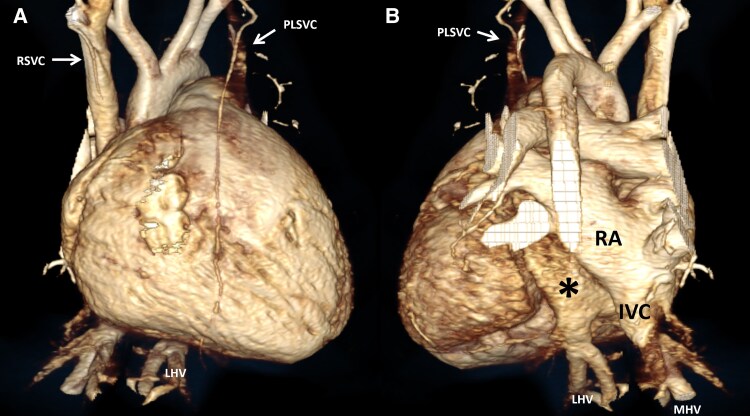
Three-dimensional (3D) volume-rendered CT images from anterior (*A*) and posterior (*B*) views demonstrate the left hepatic vein draining into the coronary sinus (star) and a persistent left superior vena cava. The middle and right hepatic veins drain into the inferior vena cava and right atrium at their normal anatomical locations. RSVC, right superior vena cava; PLSVC, persistent left superior vena cava; MHV, middle hepatic vein; LHV, left hepatic vein; RA, right atrium; *(star): coronary sinus; IVC, inferior vena cava; RHV, right hepatic vein.

Persistent left superior vena cava (PLSVC) is the most common thoracic vascular anomaly. The prevalence of PLSVC ranges from 0.2% to 3% in the general healthy population. In patients with congenital heart disease, its prevalence ranges from 1.3% to 11%. In up to 90% of cases, a right superior vena cava accompanies the PLSVC, a condition known as double SVC.^12^ The persistent left superior vena cava originates from the bifurcation of the left internal jugular and subclavian veins and extends to the coronary sinus. It is generally an isolated anomaly and does not impair haemodynamics.^13^ PLSVC usually drains into the right atrium via a dilated coronary sinus (CS) (80–92%), but in approximately 10% to 20% of cases, drainage into the left atrium (LA) can also be observed.^14^

Regarding the clinical context, chest pain in a 7-year-old patient is unusual and rarely of primary cardiac origin. However, in this particular case, it served as a critical clinical indicator of right heart strain. The massive left-to-right shunt caused by the combination of the sinus venosus ASD, PAPVR, and the anomalous LHV drainage led to significant right ventricular volume overload. We consider that the resulting increased right ventricular wall tension and relative subendocardial ischaemia likely manifested as atypical chest pain. This pathophysiology clearly links the complex anatomical findings to the patient's unusual symptomatic presentation, which ultimately prompted the initial investigations.

In conclusion, the frequency of observing variations and anomalies is increasing daily, owing to increased access to hospitals and advancements in diagnostic investigations. Although drainage of the left hepatic vein into the coronary sinus is generally a silent vascular anomaly detected incidentally, in our case, the accompanying anomalies (ASD, partial anomalous pulmonary venous return) increased the left-to-right shunt, thereby augmenting the right heart's workload and pulmonary circulation, which led to the aforementioned clinical symptoms and haemodynamic impairment.

Finally, we aimed to illustrate the summary of these coronary anomalies, whose coexistence is extremely rare, within the case study using a diagram (*Figure 4*).

**Figure 4:**
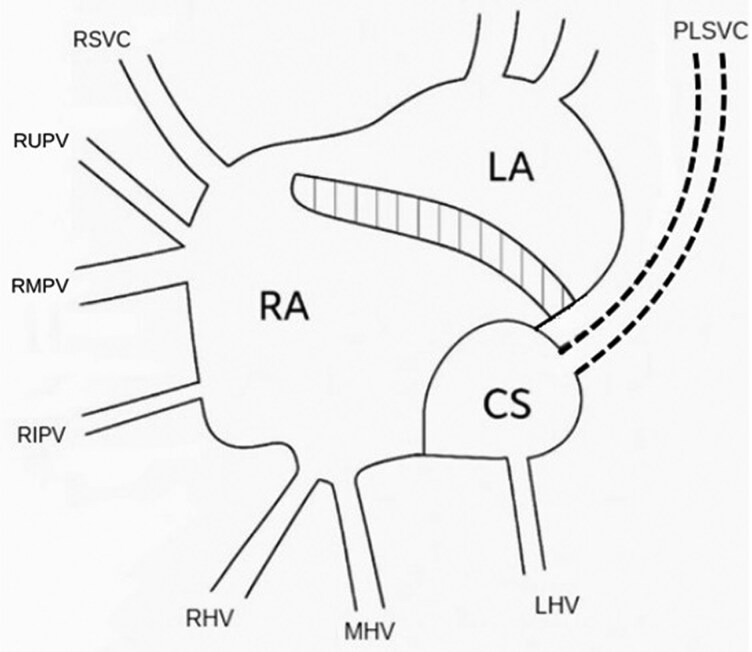
Schematic illustration depicting all the anomalies in a single diagram. RMPV, right middle pulmonary vein; RUPV, right upper pulmonary vein; PLSVC, persistent left superior vena cava; RA, right atrium; CS, coronary sinus; RHV, right hepatic vein; MHV, middle hepatic vein; LHV, left hepatic vein.

## Data Availability

The data underlying this article are available in the article.

## Lead author biography

Dr. Ömer Baran YAMAN received his medical degree from Ankara University Faculty of Medicine and is currently a radiology resident at Kayseri City Training and Research Hospital in Kayseri, Türkiye.

## Notes

**Consent:** Written informed consent was obtained from the patient’s parents for the publication of this case report and accompanying images.
